# Long‐Term Outcomes of Radiofrequency Ablation for Benign Thyroid Nodules of Different Sizes: Implications of Regrowth and New Growth

**DOI:** 10.1002/kjm2.70089

**Published:** 2025-08-11

**Authors:** Chia‐Yin Lu, An‐Ni Lin, Cheng‐Kang Wang, Pi‐Ling Chiang, Chen‐Kai Chou, Sheng‐Dean Luo, Yueh‐Sheng Chen, Chih‐Ying Lee, Jung‐Hwan Baek, Hsiu‐Ling Chen, Wei‐Che Lin

**Affiliations:** ^1^ Department of Diagnostic Radiology Kaohsiung Chang Gung Memorial Hospital and Chang Gung University College of Medicine Kaohsiung Taiwan; ^2^ Department of Diagnostic Radiology Kaohsiung Municipal Fong Shan Hospital – Under the Management of Chang Gung Medical Foundation Kaohsiung Taiwan; ^3^ Department of Diagnostic Radiology Kaohsiung Municipal Ta‐Tung Hospital Kaohsiung Taiwan; ^4^ Division of Endocrinology and Metabolism, Department of Internal Medicine Kaohsiung Chang Gung Memorial Hospital Kaohsiung Taiwan; ^5^ Department of Otolaryngology Kaohsiung Chang Gung Memorial Hospital Kaohsiung Taiwan; ^6^ Department of Radiology and Research Institute of Radiology College of Medicine, Asan Medical Center, University of Ulsan Seoul Korea; ^7^ School of Medicine, College of Medicine National Sun Yat‐Sen University Kaohsiung Taiwan

**Keywords:** goiter, neoplasm recurrence, radiofrequency ablation, treatment outcome

## Abstract

Radiofrequency ablation is an effective treatment for benign thyroid nodules. Since initial nodule volume may impact the efficacy of radiofrequency ablation, this study evaluated its long‐term outcomes across varying nodule sizes, focusing on regrowth, new growth, and clinical management implications. This retrospective study included 160 patients who underwent thyroid radiofrequency ablation for benign thyroid nodules at a Taiwanese tertiary center between July 2016 and April 2018. Patients were classified into three groups based on nodule size: small (< 10 mL), medium (10–30 mL), and large (> 30 mL). Treatment efficacy was assessed over a period of up to 5 years, focusing on volume reduction rate, regrowth, residual volume, and new growth. The initial ablation rate of all benign thyroid nodules was 99.46%. After the 5‐year follow‐up, the volume reduction rate was 92.96%. The small nodule group demonstrated the highest volume reduction rate. The incidence of increased residual vital volume was 3.57%. The overall regrowth rate was 9.82%, with a mean time to regrowth of 2.8 years. No nodules required retreatment due to regrowth. New growth was observed in 22.32% of patients, with the highest incidence in the large nodule group (34.29%). Radiofrequency ablation is effective in the long‐term management of benign thyroid nodules across various sizes, achieving substantial volume reduction rate with minimal complications. For larger nodules, monitoring for new growth warrants increased attention and may serve as a critical parameter indicative of recurrence and the potential need for retreatment.

**Abbreviations**
BTN: benign thyroid nodule
IAR: initial ablation ratio
NO.: number
RFA: radiofrequency ablation
SD: standard deviation
VRR: volume reduction rate
Vt: total volume
Vv: residual vital volume

## 1 Introduction

Radiofrequency ablation (RFA) has emerged as an effective alternative to surveillance or surgery for benign thyroid nodules (BTNs), preserving thyroid function and reducing the need for lifelong medication [1, 2, 3, 4]. By generating frictional heat that induces coagulative necrosis and apoptosis, RFA is endorsed by multiple national guidelines as a primary treatment for BTNs [5, 6, 7, 8, 9, 10, 11, 12, 13, 14, 15, 16, 17, 18]. Beyond BTNs, RFA has also been applied to autonomous functional thyroid nodules, papillary microcarcinoma, recurrent thyroid cancer, and even extrathyroidal lesions such as parotid tumors and hemangiomas [17, 19].

Previous studies report volume reduction rates (VRRs) of 70%–94% [6, 7, 8, 9, 10, 11, 12, 13, 14, 20]. In Taiwan, where BTNs affect 19.4% of men and 33.6% of women [15], effective therapies are essential. Since the establishment of Taiwan's formal thyroid RFA consensus in 2022 [16], RFA has demonstrated strong local efficacy. For instance, a 6‐month study of 826 nodules achieved a VRR of 73.2% [15], and a 2‐year analysis of 153 nodules of varying sizes reported a VRR of 85.5%, with regrowth in 3.9% and new growth in 18.9% of cases [6]. Although evidence suggests that residual volume increases and regrowth rates rise with longer follow‐up [6, 8, 11, 21], long‐term data on regrowth and new growth rates remain limited.

Regrowth, defined as an increase of more than 50% in total nodule volume compared to the smallest recorded volume [6], is a critical parameter for evaluating RFA efficacy and the need for retreatment [16, 22, 23, 24]. It is influenced by initial nodule volume, vascularity, energy delivered, and residual vital volume (Vv) [6, 8, 21]. Vv has also been identified as a critical parameter for assessing retreatment needs [8]. New growth, defined as an increase in new vital volume not detected in early follow‐up ultrasounds (less than 6 months) [6], may serve as an early indicator of regrowth and recurrence, potentially guiding the decision for RFA retreatment. The size of the nodule appears to affect both regrowth and new growth rates [6], underscoring the importance of evaluating RFA efficacy by stratifying nodules according to their size.

This study aims to evaluate the long‐term outcomes of RFA across varying nodule sizes, focusing on regrowth, new growth, and the subsequent implications for clinical management.

## 2 Materials and Methods

### 2.1 Compliance With Ethical Standards

This study was conducted in compliance with ethical standards. Data were retrospectively collected from patients who underwent RFA for BTNs at a tertiary referral medical center in Taiwan from July 2016 to April 2018. The study was approved by the Institutional Review Board of the Chang Gung Medical Foundation (protocol number 202401123B0). Written informed consent was obtained from all patients prior to the RFA procedure. Given the retrospective nature of the study, the requirement for informed consent for publication was waived.

### 2.2 Patient Acquisition and Pre‐Ablation Assessment

Patients typically sought RFA due to cosmetic concerns or symptoms such as neck pain or dysphagia related to enlarged thyroid nodules. RFA was considered an alternative for patients unwilling to undergo surgery due to concerns about scarring or complications.

**FIGURE 1:**
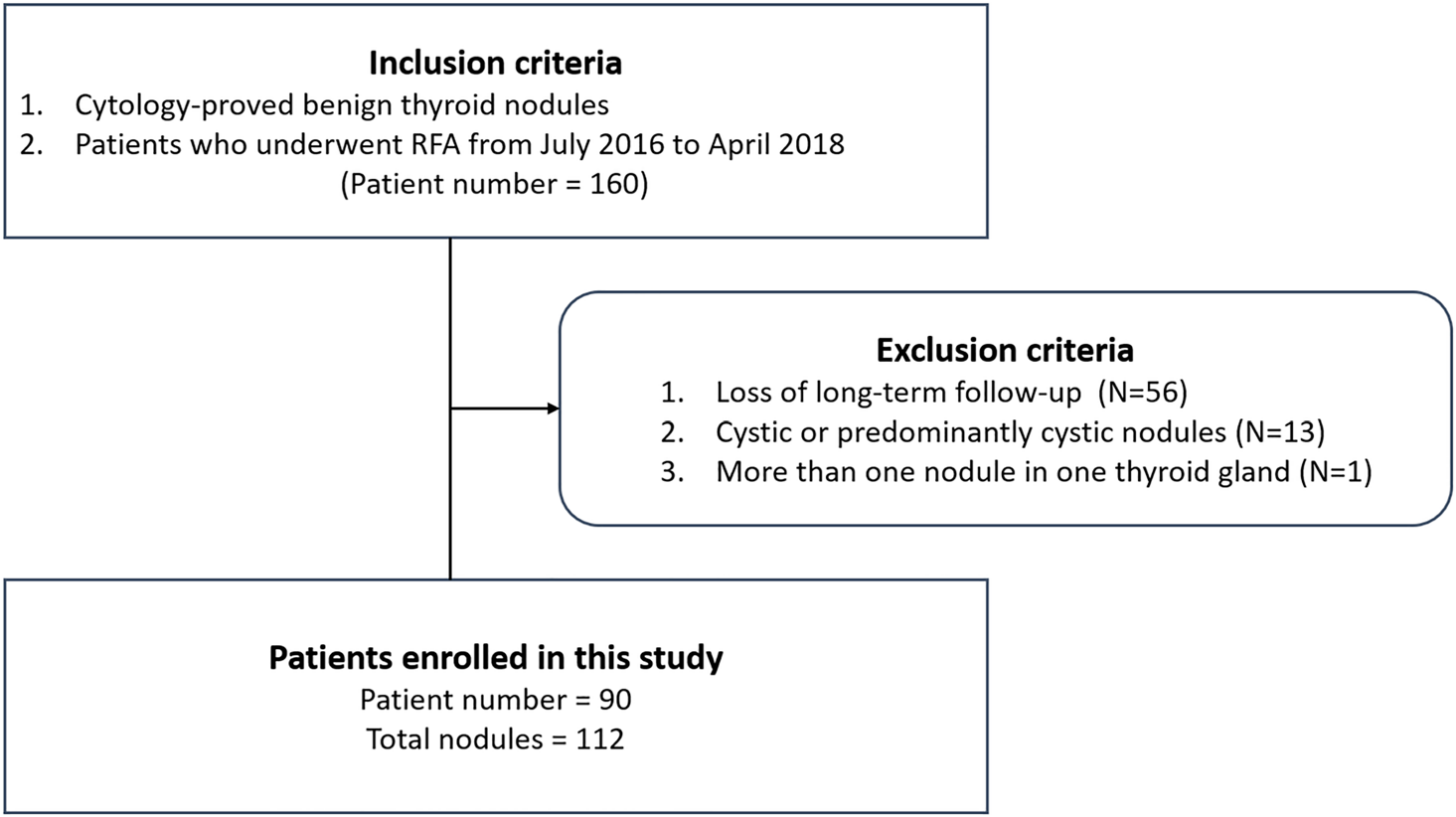
Flowchart of patient enrollment and exclusion. The inclusion criteria were: (1) cytological confirmation of benignity; and (2) receipt of RFA at our institution between July 2016 and April 2018. The exclusion criteria were: (1) loss of long‐term follow‐up; (2) cystic or predominantly cystic nodules; (3) presence of multiple ipsilateral nodules. Consequently, data from 90 patients with a total of 112 nodules were analyzed. N, patient number; RFA, radiofrequency ablation.

The inclusion criteria were: (1) cytological confirmation of benignity, defined as either two independent benign fine‐needle aspiration (FNA) results or a single benign FNA result accompanied by benign ultrasonographic features, specifically an isoechoic spongiform architecture or a partially cystic nodule with intra‐cystic comet‐tail artifacts; and (2) receipt of RFA at our institution between July 2016 and April 2018. A total of 160 patients met these criteria and were initially included in the study (Figure 1). The exclusion process involved three steps: (1) 56 patients were excluded due to loss of long‐term follow‐up (defined as at least 2 years); (2) 13 patients were excluded due to the presence of cystic or predominantly cystic nodules. Predominantly cystic nodules are characterized by a fluid component of 51%–90%, while cystic nodules have a fluid component exceeding 90%. This exclusion was made to avoid confounding effects from ethanol sclerotherapy and to account for the nature of these nodules, which could significantly impact RFA efficacy; (3) one patient was excluded due to the presence of multiple ipsilateral nodules, as merging of nodules in a single thyroid lobe during follow‐up could affect calculation accuracy. To clarify, patients with two nodules in contralateral lobes were not excluded. Consequently, data from 90 patients with a total of 112 nodules were analyzed.

To address the variability in initial nodule size and the response to RFA, nodules were categorized into three groups based on baseline volume assessed via sonographic evaluation: (1) small (< 10 mL); (2) medium (10–30 mL); and (3) large (> 30 mL).

Prior to RFA, each patient underwent a comprehensive assessment including medical history, cosmetic and symptom scores, thyroid function tests (TSH, T3, free T4), and ultrasound evaluation of the thyroid nodule volume and cervical lymph nodes. For larger nodules, CT or MRI was used to assess potential intrathoracic extension. Additionally, laryngoscopy was performed by an otorhinolaryngologist to evaluate vocal cord function.

### 2.3 Ablation Techniques

Patients were positioned supine with their necks fully extended and were administered parecoxib 40 mg IV, if not contraindicated, to reduce procedural discomfort. Local anesthesia was administered using a mixture of lidocaine, epinephrine, and sodium bicarbonate. All RFA procedures were performed on an outpatient basis by a single radiologist with over 10 years of experience. The procedure utilized RF generators (RF150 and RF300, Apro‐Korea, Gunpo, Korea) and straight‐type modified internally cooled electrodes with active tip lengths of 5, 7, and 10 mm (Well‐Point RF Electrode, STARmed, Goyang, Korea; CoATherm electrode, Apro‐Korea). The electrode tip size was selected based on tumor size and surrounding structures. Techniques employed included the trans‐isthmic approach, moving‐shot technique, and hydrodissection. Ablation was ceased when the nodule exhibited transient hyperechoic zones.

Complications were monitored and managed during and immediately after the procedure. Post‐procedural laryngoscopy was performed to assess vocal cord function before patient discharge.

### 2.4 Follow‐Up Assessment

Follow‐up visits were scheduled at 1, 3, and 6 months post‐RFA, and annually thereafter. Nodule volume, cosmetic scores, and symptom scores were evaluated using the same methods before and after ablation. Nodule volume was calculated using ultrasound with the formula V = πabc/6 (V, volume; a, the largest diameter; b and c, the other two perpendicular diameters in centimeters).

To assess RFA efficacy and recurrence, several parameters were defined. The VRR was calculated using the equation: ([initial volume—final volume] × 100)/initial volume. The initial ablation ratio (IAR) was determined as the ratio between the ablated volume (Va) and the total volume (Vt), using the formula: IAR = (Va/Vt) × 100, reflecting technique efficacy. The Va represents the hypoechoic area without vascularity on ultrasound. The Vt of a nodule is divided into Va and Vv portions. Vv denotes the incompletely treated vital nodule volume observed on follow‐up ultrasounds within 3 months.

**FIGURE 2:**
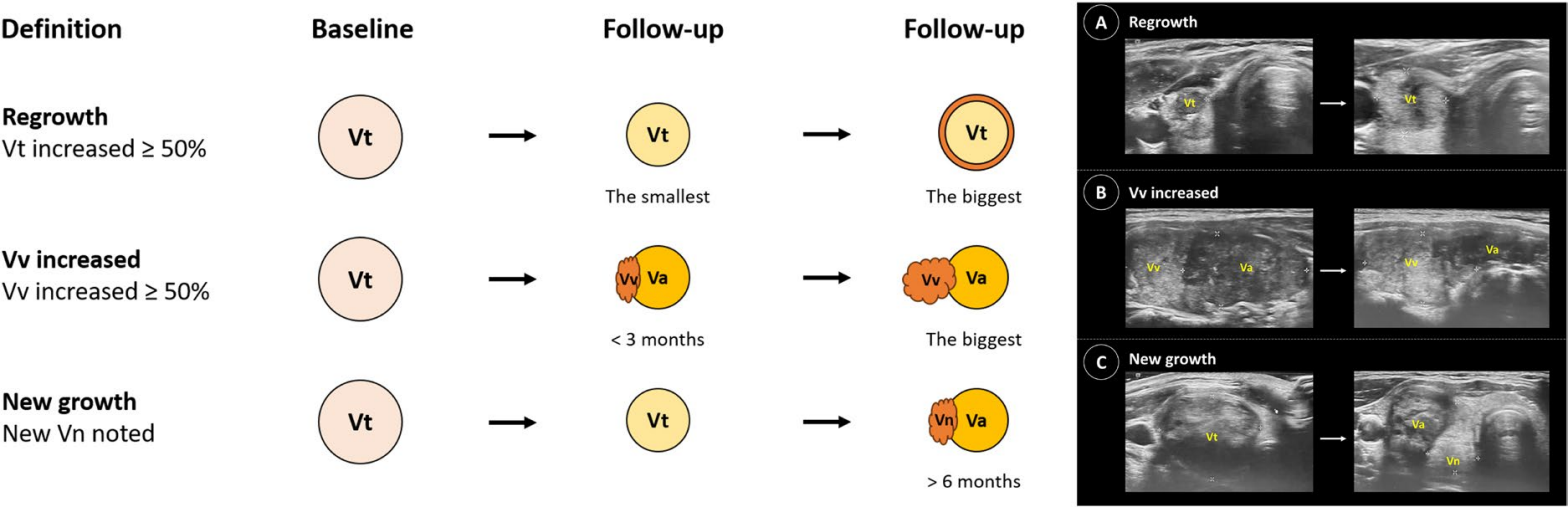
Definition of volume assessments. Ultrasound images illustrating residual vital volume (Vv) increase and new growth. (A) Regrowth: Total volume (Vt) increased more than 50% compared to the smallest volume previously reported on ultrasonography. (B) Residual vital volume (Vv) increased more than 50% compared to the previously reported smallest volume. The Vv represents the incompletely treated vital nodule noted at less than 3 months. (C) New growth: volume not detected in early follow‐up ultrasonography; however, an isoechoic lesion was noted after 6 months.

New growth was defined as an increase in new vital volume not detected in early follow‐up ultrasounds (less than 6 months) [6]. The increase in Vv was defined as more than a 50% increase in Vv compared to the smallest recorded volume. Regrowth was defined as more than a 50% increase in Vt compared to the smallest recorded volume. Definitions of these parameters are illustrated in Figure 2.

Additional RFA was considered under two circumstances. First, in accordance with international guidelines and the Taiwan Thyroid RFA Consensus, retreatment was indicated when the VRR remained < 50% [22, 25] or < 30% [24], when regrowth was identified [16, 22, 23, 24], or persisted symptoms [16, 22, 23, 24].

For nodules with a baseline maximal diameter > 5 cm, particularly intrathoracic goiters, we routinely pre‐planned a second, staged RFA session 6 months after the initial procedure when the patient consented. These staged sessions were regarded as part of the index therapy and were therefore not classified as “retreatment” in our analysis.

### 2.5 Statistical Analysis

Statistical analyses were performed using SPSS version 22 (SPSS Inc., Chicago, IL, USA). Comparisons of demographic characteristics and sonographic results among subgroups were conducted. Changes in volume, frequency, and timing of Vv increase, regrowth, and new growth were analyzed and compared across the three groups. Continuous variables were compared using an analysis of covariance (ANCOVA) model, with age and gender as covariates. Categorical data were analyzed using the Chi‐Square test. Data are presented as mean ± standard deviation (SD) or number of nodules (percentage). The 95% CIs for means were calculated using the Student's t‐distribution, and 95% CIs for proportions were calculated using the Clopper‐Pearson exact method. Statistical significance was set at *p* < 0.05.

## 3 Results

### 3.1 Demographic Characteristics

The characteristics of the patients and nodules are presented in Table 1. The study cohort comprised 90 patients (77 females and 13 males) with a mean age of 44.22 years. A total of 112 thyroid nodules were evaluated. Of the patients, 68 had unilateral nodules and 44 had bilateral nodules. The mean nodule volume was 29.19 mL. Nodules were categorized into three volume groups: small (< 10 mL) with 41 nodules (36.6%), medium (10–30 mL) with 36 nodules (32.1%), and large (> 30 mL) with 35 nodules (31.3%).

**TABLE 1:** Patients' demographic data and nodule characteristics.

Variables	Characteristics
No. of Patients	90
Gender (Female: Male)	77: 13
Age (years)	44.22 ± 11.14
No. of nodules	112
Unilateral nodule	68
Bilateral nodules	44
Volume (mL)	29.19 ± 38.33
Volume < 10 mL, *n* (%)	41 (36.6%)
Volume 10–30 mL, *n* (%)	36 (32.1%)
Volume > 30 mL, *n* (%)	35 (31.3%)
Nodule structure, *n* (%)	
Solid	42 (37.5%)
Predominantly solid	70 (62.5%)
Thyroid function test (baseline)	
Serum TSH (uIU/mL)	1.20 ± 0.84
Serum T4 (ng/dL)	1.33 ± 0.70
Serum T3 (ng/dL)	98.28 ± 19.04
Cosmetic score (baseline)	2.16 ± 1.04
Symptomatic score (baseline)	1.11 ± 1.08

*Note*: Values are presented as mean ± standard deviation (SD), number of nodules (percentages).

Abbreviation: No., number.

In terms of nodule composition, 42 nodules (37.5%) were solid, while 70 nodules (62.5%) were predominantly solid, defined as having a fluid component between 11% and 50%. Baseline thyroid function tests were within normal limits. Baseline cosmetic and symptom scores were 2.16 and 1.11, respectively.

### 3.2 Treatment Outcomes by Nodule Size and VRR

Table 2 presents changes in nodule size and corresponding VRR values across the three groups. There were no significant differences in age or sex among the groups. All groups exhibited a decrease in nodule size over time, with significant intergroup differences (*p* < 0.001) observed over the longer follow‐up periods.

**TABLE 2:** Nodule volume changes according to the initial nodular size.

Characteristics	Total	Small	Medium	Large	*p*
No. of nodules	112	41	36	35	
Nodule volume (mL)					
Baseline	29.19 ± 38.33	4.01 ± 2.99^ab^	17.71 ± 5.59^ac^	70.50 ± 45.76^bc^	< 0.001*
6 months	9.36 ± 19.80	1.01 ± 1.48^b^	6.00 ± 3.57^c^	24.42 ± 32.10^bc^	< 0.001*
12 months (1 year)	5.81 ± 8.01	0.51 ± 1.09^ab^	3.98 ± 3.37^ac^	14.11 ± 9.54^bc^	< 0.001*
24 months (2 years)	4.96 ± 7.91	0.44 ± 1.02^b^	2.83 ± 2.41^c^	12.57 ± 10.76^bc^	< 0.001*
36 months (3 years)	4.06 ± 6.15	0.60 ± 1.17^b^	1.74 ± 1.75^c^	9.70 ± 7.75^bc^	< 0.001*
48 months (4 years)	2.62 ± 4.64	0.50 ± 1.27^b^	1.52 ± 2.06^c^	6.24 ± 6.77^bc^	< 0.001*
60 months (5 years)	2.27 ± 5.17	0.11 ± 0.13^b^	1.54 ± 2.25^c^	9.67 ± 9.62^bc^	< 0.001*
VRR (%)					
6 months (95% CI)	71.11 ± 15.59 (67.61–74.61)	74.24 ± 21.21 (67.76–80.72)	67.67 ± 15.16 (62.01–73.33)	70.67 ± 14.18 (65.85–75.49)	0.386
12 months (1 year) (95% CI)	81.25 ± 15.55 (78.16–84.34)	86.40 ± 15.73 (81.26–91.54)	78.14 ± 16.98 (72.64–83.64)	78.56 ± 12.32 (74.55–82.57)	0.051
24 months (2 years) (95% CI)	85.32 ± 13.39 (82.44–88.20)	88.86 ± 15.29 (83.98–93.74)	84.42 ± 13.36 (80.10–88.74)	82.44 ± 10.46 (78.84–86.04)	0.212
36 months (3 years) (95% CI)	88.05 ± 12.49 (85.15–90.96)	90.06 ± 15.08 (85.07–95.06)	90.04 ± 9.60 (87.02–93.06)	84.15 ± 11.88 (80.20–88.10)	0.193
48 months (4 years) (95% CI)	90.54 ± 11.79 (86.88–94.19)	91.45 ± 13.69 (86.49–96.41)	90.57 ± 12.84 (86.05–95.09)	89.43 ± 8.61 (86.47–92.39)	0.782
60 months (5 years) (95% CI)	92.96 ± 9.74 (88.98–96.94)	96.28 ± 3.93 (94.84–97.72)	91.21 ± 13.65 (85.84–96.57)	87.36 ± 10.89 (82.96–91.77)	0.093
Initial ablation ratio (IAR, %)	99.46 ± 3.84	99.87 ± 0.84	98.93 ± 6.41	99.54 ± 2.11	0.596
No. of Vv increase (%) (95% CI)	4 (3.57%) (0.98%–8.89%)	1 (2.44%) (0.06%–12.86%)	1 (2.78%) (0.07%–14.53%)	2 (5.71%) (0.70%–19.16%)	0.710
Retreated (*n*, %)	2 (1.79%)	1 (2.44%)	0 (0.00%)	1 (2.78%)	0.612
No. of regrowth (*n*, %) (95% CI)	11 (9.82%) (5.01%–16.89%)	3 (7.32%) (1.54%–19.92%)	4 (11.11%) (3.11%–26.06%)	4 (11.43%) (3.20%–26.74%)	0.606
Timing of regrowth (month)	33.82 ± 14.01	28.00 ± 13.86	42.00 ± 12.00	30.00 ± 15.49	0.633
Retreated (*n*, %)	0	0	0	0	
No. of new growth (%) (95% CI)	25 (22.32%) (15.00%–31.16%)	5 (12.20%) (4.08%–26.20%)	8 (22.22%) (10.12%–39.15%)	12 (34.29%) (19.13%–52.21%)	0.070
New growth (mL)	1.13 ± 1.72	0.42 ± 0.31	1.64 ± 2.68	1.09 ± 1.21	0.523
Timing of recurrence (month)	25.60 ± 14.18	23.00 ± 8.06	22.25 ± 18.25	28.92 ± 13.41	0.458
Retreated (*n*, %)	8 (7.14%)	0	4 (11.43%)	4 (11.11%)	0.083
Complications and side effects					
Adverse events (*n*, %)					
Major complications					
Voice change	3 (2.68%)	2 (4.88%)	0	1 (2.78%)	0.416
Minor complications					
Rupture	0	0	0	0	
Blepharoptosis	1 (0.89%)	0	0	1 (2.78%)	0.330

*Note*: Values are presented as mean ± standard deviation or number of nodules (percentages) (95% CI); The *p*‐values presented here compare the parameters among the three subgroups (small, medium, and large) using ANCOVA with age and sex as covariates. Statistically significant *p*‐values are indicated with an asterisk (*). Superscript ‘a’, significant differences between the small and medium groups in the post hoc analysis. Superscript ‘b’, significant differences between the small and large groups. Superscript ‘c’, significant differences between the medium and large groups.

Abbreviations: No., number; VRR, volume reduction rate; Vv, vital volume.

**FIGURE 3:**
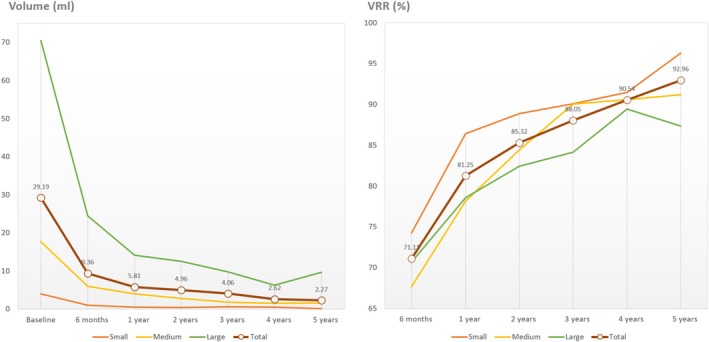
Volume size and volume reduction ratio (VRR) in subgroups. The changes of volume size and volume reduction rate at each follow‐up point after RFA. The values on the graph represent the average of total nodules at the corresponding time points.

Figure 3 illustrates trends in volume reduction and VRR over the 5‐year follow‐up period. Each group demonstrated a significant volume reduction, with the most substantial decrease occurring within the first year. At 5 years, the VRR for the small, medium, and large groups was 96.3% (95% CI 94.84–97.72), 91.2% (95% CI 85.84–96.57), and 87.4% (95% CI 82.96–91.77), respectively, yielding an overall VRR of 93.00% (95% CI 88.98–96.94). Smaller nodules exhibited numerically the highest VRR, followed by medium and large nodules.

### 3.3 Treatment Outcomes by Regrowth, Residual Volume, and New Growth

**FIGURE 4:**
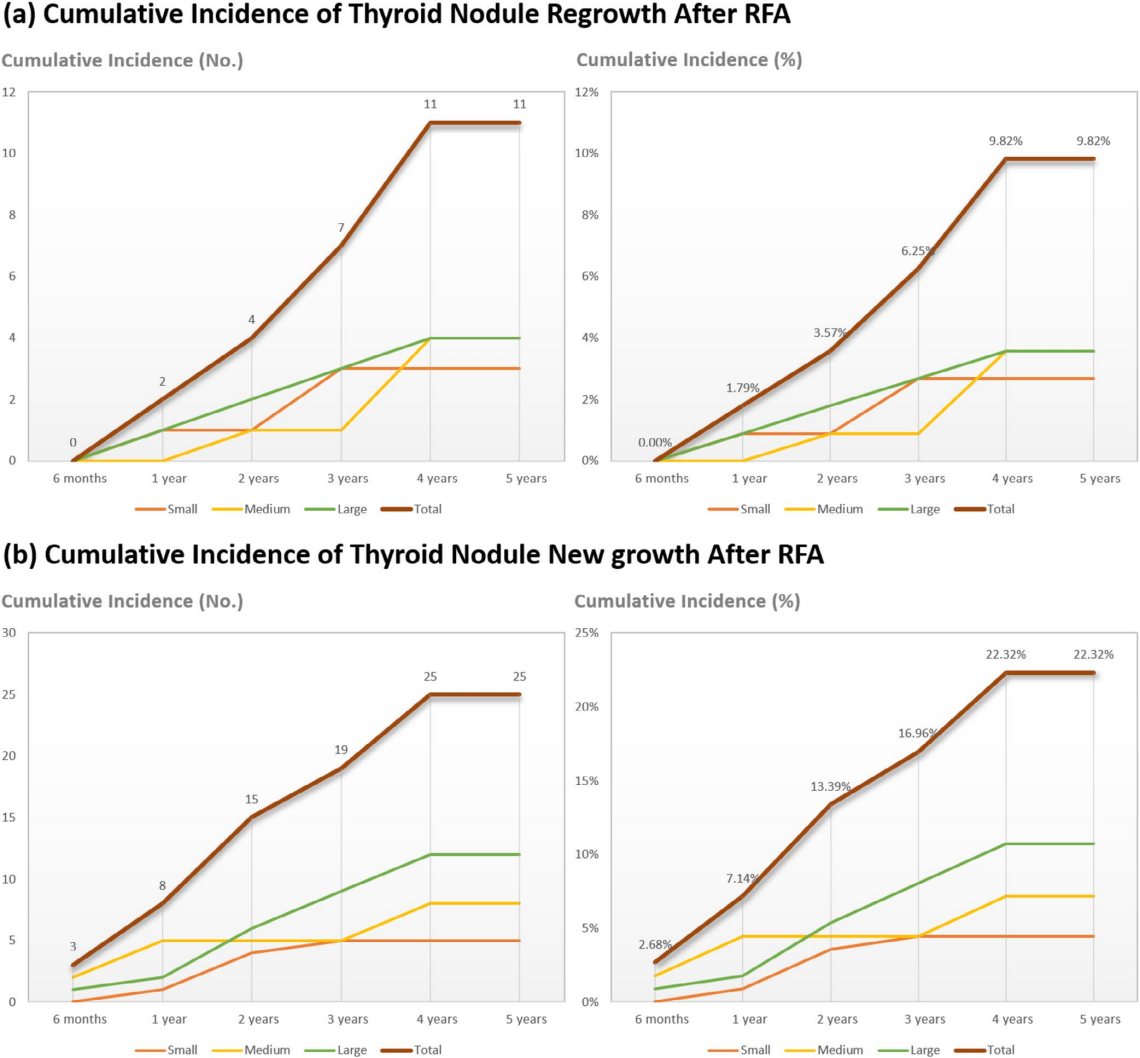
Cumulative incidence of thyroid nodule regrowth (A) and new growth (B) after RFA in 5‐year follow‐up. Cumulative incidence of thyroid nodule regrowth (A) and new growth (B) observed during follow‐up after RFA.

Table 2 shows the IAR for all groups, approaching 98% across the board, indicating high ablation efficacy. Regrowth, defined as an increase of more than 50% in total nodule volume compared to the smallest recorded volume, occurred in 11 nodules: three in the small group, four in the medium group, and four in the large group (*p* = 0.606). The highest cumulative incidence of regrowth was observed in the medium and large groups (3.57% each), followed by the small group (2.68%) (Figure 4A). The earliest regrowth was noted in the small group (28 months), followed by the large (30 months) and medium groups (42 months), with an average regrowth time of 33.82 months. Regrowth instances were recorded within the 4‐year follow‐up period (Table 3). No nodules with regrowth required additional RFA or delayed surgery.

**TABLE 3:** Nodule volume changes according to the follow‐up period.

Variables	Baseline	6 months	1 year	2 years	3 years	4 years	5 years
No. nodules	112	97	98	83	71	42	23
Vt (mL)	29.19 ± 38.33	9.36 ± 19.80	5.81 ± 8.01	4.96 ± 7.91	4.06 ± 6.15	2.62 ± 4.64	2.27 ± 5.17
VRR (%) (95% CI)	—	71.11 ± 15.59 (67.61–74.61)	81.25 ± 15.55 (78.16–84.34)	85.32 ± 13.39 (82.44–88.20)	88.05 ± 12.49 (85.15–90.96)	90.54 ± 11.79 (86.88–94.19)	92.96 ± 9.74 (88.98–96.94)
No. regrowth	—	0	2	2	3	4	0
No. new growth	—	3	5	7	4	6	0
New growth (mL)	—	3.58 ± 4.10	0.94 ± 0.54	1.28 ± 1.40	0.40 ± 0.36	0.39 ± 0.38	—

*Note*: Values are presented as mean ± SD (95% Confidence interval) or n (number of nodules). Baseline VRR is not applicable. A dash (−) indicates no data/not applicable.

Abbreviations: No., number; VRR, volume reduction rate; Vt, vital volume.

Among the 112 nodules, 4 had Vv identified during the first 3 months of follow‐up (small, *n* = 1, 2.44%, 95% CI 0.06%–12.86%; medium, *n* = 1, 2.78%, 95% CI 0.07%–14.53%; large, *n* = 2, 5.71%, 95% CI 0.70%–19.16%). Two of these nodules (one from the small group and the other one from the large group) underwent retreatment with RFA.

New growth, defined as an increase in nodule volume not detected in the initial 6‐month follow‐up, occurred in 25 nodules (22.32%, 95% CI 15.00%–31.16%): small group (12.20%, 95% CI 4.08%–26.20%), medium group (22.22%, 95% CI 10.12%–39.15%), and large group (34.29%, 95% CI 19.13%–52.21%). There was a trend toward a higher proportion of new growth in larger nodules (*p* = 0.07). The average time to new growth was 25.6 months, with no significant differences among groups. Eight nodules (small, *n* = 4; large, *n* = 4) underwent repeated RFA. No nodules with new growth required delayed surgery. New growth was detected within the 4‐year follow‐up, with no significant variations among groups (Table 3). The highest cumulative incidence of new growth was noted in the large group (10.71%), followed by the medium group (7.14%) and the small group (4.46%) (Figure 4B).

### 3.4 Complications

Table 2 summarizes complications observed across subgroups. All patients with complications recovered without sequelae. Out of 112 nodules, 4 (3.57%) experienced complications: small group (2 cases, 4.88%) and large group (2 cases, 5.56%), with no significant differences among groups. Voice change was the most common complication (*n* = 3, 2.68%, recovered within 45–70 days), followed by blepharoptosis (*n* = 1, 0.89%). In the large group, voice change and blepharoptosis were each observed in one case (2.78%). In the small group, voice change was noted in two cases (4.88%).

## 4 Discussion

### 4.1 Summary

RFA is a safe and effective method for treating BTNs, resulting in a clinically significant reduction in the volume of BTNs across various sizes, with this effect sustained for at least 5 years. Our study demonstrated a significant VRR of 92.96% and a minimal regrowth rate of 9.82% over the 5‐year period. The most substantial therapeutic effect was observed within the first year, with steady volume reductions thereafter. New growth was observed in 22.32% of nodules, with 7.14% undergoing retreatment, predominantly in larger nodules (*p* = 0.07). Both regrowth and new growth were primarily observed within the first 4 years, with no new cases emerging in the fifth year. Three cases with voice change resolved within a few months and did not vary by nodule size.

### 4.2 Long‐Term Efficacy of RFA in the Three Groups

Among the three size categories, the small group achieved numerically the highest VRR (Figure 3). Despite initially less favorable outcomes in the first 2 years as reported in our 2‐year follow‐up study [6], long‐term follow‐up indicated that smaller nodules consistently maintained superior VRR, consistent with other research findings [26]. Previous studies suggest that the initially lower VRR for smaller nodules may be attributed to the relatively larger ablation margins [15]. Larger nodules, including those with intrathoracic goiter, may experience uneven heat distribution and anatomical constraints (bottleneck hypothesis) [4], leading to suboptimal VRR. Nodule size remains a crucial factor influencing ablation efficacy, with RFA showing greater success in smaller nodules [13, 27]. Our study, despite starting with the largest initial nodule volume (29.19 mL), achieved a commendable VRR of 92.96%, as compared with other long‐term studies (Table 4) [1, 6, 8, 11, 12, 26, 28]. Other factors influencing VRR, such as energy delivery, peripheral flow blockage, and ablation margins, have been discussed in the literature [29].

**TABLE 4:** Comparison of the regrowth rate between our study and other studies.

Study	Nodule number	Initial volume (mL)	Nodule characteristic (%)	VRR (%)					Follow up period (m)	Regrowth rate (%)					Regrowth timing (m)
				1 year	2 years	3 years	4 years	5 years		1 year	2 years	3 years	4 years	5 years	
Our (2022)	153	28.44 ± 37.97	45.1 (solid), 54.9 (pred‐solid)	79.21	85.53				24	—	3.92	—	—	—	16.71 ± 9.46
Our (now)	112	29.19 ± 38.33	37.5 (solid), 62.5 (pred‐solid)	81.25	85.32	88.05	90.54	92.96	67.8	1.79	3.57	6.25	9.82	9.82	33.82
Sim 2017 (Korea)	54	14.0 ± 12.70	70.4 (solid), 20.4 (mixed), 9.3 (cystic)	73.61		69.60		73.50	39.4	3.85	—	18.92	—	25.00	39.9 ± 17.5
Kim 2022 (Korea)	90	14.3 ± 16.20	88.89 (solid), 11.11 (cystic)	76.6	84.1	85.4	87.4	74.5	66.6	1.60	7.30	8.50	5.00	21.10	—
Yan 2020 (China)	88	10.49 ± 14.87	—				94.29		35.52	6.82	14.77	18.18	20.45	—	24.33 ± 12.42
Yan 2020 (China)	206	10.09 ± 12.90	69.9 (solid)	84.90	89.29				22.50	—	12.62	—	—	—	20.77 ± 12.03
Bernardi 2020 (Italy)	216	17.20 (median)	75 (solid), 19 (pred‐solid), 5 (pred‐cystic), 1 (cystic)	72.4	74.6	75.9	76.3	77.1	60	0.00	6.10	7.90	14.70	17.20	—
Bernardi 2021 (Italy)	82	11.30 (median)	44 (solid), 35 (pred solid), 21 (pred cystic)	76	76	77	79	79	60	—	—	—	—	23.17	—

*Note*: This table presents the long‐term follow‐up results of our study and other long‐term studies on benign thyroid nodules after RFA, including nodule number, initial volume (presented as mean ± SD or median), nodule characteristics, volume reduction rate by year, mean follow‐up periods, regrowth rate, and regrowth timing. m (month).

Among the five studies with regrowth rate data at 5 years [8, 11, 30, 31], Bernardi et al. (2020) [30] reported the highest number of nodules (*n* = 216), followed by our study (*n* = 112). All studies reported VRRs of at least 73.5%, with generally increasing VRRs over longer follow‐up durations. A previous systematic review indicated pooled VRR values starting at 76.9% and rising to 92.2% by the final follow‐up [12] This suggests that long‐term VRR can improve from 70% to 90% with extended follow‐up. The VRR plateau was observed between one and 3 years, followed by continued volume reduction, which is consistent with Bernardi et al.'s findings. Our study's consistently high VRR further underscores its efficacy in sustaining long‐term volume reduction.

### 4.3 IAR and Residual Vital Volume Increases

The IAR is a predictor of both volume reduction and the likelihood of retreatment [8, 30, 32]. Our study recorded an average IAR of 99.46, with the lowest in the medium group at 98.93. A high IAR is associated with satisfactory VRR, irrespective of initial nodule size [6].

The increase in Vv is critical for predicting regrowth, as regrowth is only detectable when the increase in Vv exceeds the decrease in Va [8]. Sim et al. proposed Vv increase as a parameter for early recurrence detection [6, 31]. Although long‐term data on Vv increase is limited, our findings indicate that there was no significant difference in the rate of Vv increase among the large, medium, and small groups (*p* = 0.71). Our study demonstrated a Vv increase rate of 3.57% (95% CI 0.98%–8.89%) at 5‐year follow‐ups, which is lower than the rates reported in other studies of 25% and 21.1% [8, 31]. Vv increase typically precedes Vt increase [8], consistent with our findings and those of other studies [31].

### 4.4 Regrowth and New Growth Rates

Current treatments for BTNs include various thermal and chemical ablation techniques. Thermal ablation methods such as RFA, microwave ablation, laser ablation, and high‐intensity focused ultrasound (HIFU) have been compared, with RFA often demonstrating higher VRR compared to microwave ablation [13] and a lower regrowth rate compared to laser ablation [12, 28] Yan et al. reported that the initial volume in regrowth cases was significantly larger than in non‐regrowth cases [21] Park et al. found that nodules with an initial volume of ≥ 20 mL had a significantly higher risk of regrowth compared to smaller nodules [14] Our study did not reveal significant differences in regrowth rates among the three size groups (*p* = 0.606).

Factors contributing to regrowth include the residual vital ratio and nodule characteristics such as location and vascularity [21, 32, 33, 34]. Incomplete ablation and low energy delivery may also contribute to regrowth [27, 32]. Thorough treatment of the margin is essential to prevent marginal regrowth [31].

The earliest regrowth was detected in the small group at 28 months, likely due to the smaller nodules reaching the regrowth criterion more quickly. Larger nodules, in contrast, face greater challenges meeting the regrowth criteria. This finding also explains why the incidence of new‐growth cases exceeded that of regrowth cases in the large group (Table 2). While smaller nodules are more prone to regrowth, their clinical significance is limited due to minimal mass effects, thus rarely necessitating further treatment. Larger nodules, however, may experience symptoms even with minimal new growth. In our study, no patients required retreatment due to regrowth, although eight patients (medium group: 4; large group: 4), primarily with larger nodules, underwent retreatment for new growth after thorough discussion.

Our previous 2‐year study documented short‐term results, with a mean VRR of 85%, regrowth of 3.9%, and new growth of 18.9%. The present investigation extends follow‐up to 5 years in an expanded cohort, thereby capturing additional late events (cumulative regrowth 9.8%, cumulative new growth 22.3%), refining retreatment criteria, and confirming durable nodule control, with a 5‐year VRR of 93% (95% CI 88.98–96.94).

Studies generally show an increase in regrowth rates over time, with our study demonstrating a more modest rise from the first to fifth year compared to others. Previous studies have shown that approximately 20% of patients who undergo RFA experience regrowth within 5 years [11]. The cumulative regrowth rate in our study (9.82%) is favorable compared to other reports (17.2%–25.0%) and reflects a lower risk of regrowth, likely due to a higher IAR. Regrowth typically occurs in two peaks, at 2–4 years and after 5 years, with reported times ranging from 16.7 to 39.9 months (Table 4). This suggests that even nodules which appear stable can experience marginal regrowth and that additional RFA could be performed to achieve a higher VRR and superior long‐term results [8, 21, 31]. The absence of regrowth or new growth in the fifth year suggests disease stabilization by 4 years post‐treatment, indicating that a follow‐up period of at least 4 years is advisable.

Earlier research indicates that retreatment is more common in younger patients, those with larger nodules, cases with a lower 1‐year VRR, and cases of insufficient energy [28, 32]. Although international guidelines often recommend retreatment based on VRR, regrowth, and symptoms, they do not typically consider new growth [16, 22, 23, 24]. Our findings suggest that monitoring new growth should be an important parameter for detecting recurrence and determining the need for retreatment, particularly for larger nodules.

Our previous study reported that the new growth rate, mean timing of new growth, and volume of new growth were 18.75%, 16.9 months, and 1.92 mL [6], respectively. In comparison, the current study found these values to be 22.32% (95% CI 15.00%–31.16%), 25.6 months, and 1.13 mL. A trend toward higher prevalence was observed in larger BTN groups, consistent with our primary findings. In the previous study, no retreatment was necessary due to the relatively low volume of new growth. However, in the current study, eight patients, particularly those with larger BTNs, underwent retreatment following discussions with the patients. Previous research has shown that larger nodules are more prone to new growth, particularly with longer follow‐up periods [6, 33, 34], which supports our findings. In addition to initial nodule size, several other factors may predict new growth and should be investigated in future studies.

### 4.5 Limitations

This study has several limitations. As a retrospective, single‐center analysis, it may face challenges related to patient compliance and might not fully capture the broader Taiwanese population. The exclusion of patients with multiple nodules on one side limits the ability to assess unilateral multiple nodules. Additionally, variations in nodule characteristics across different studies may complicate direct comparisons. The reliance on grayscale ultrasound alone, rather than incorporating contrast‐enhanced techniques, may lead to underestimation of Vv and new growth nodules. The use of contrast‐enhanced ultrasound (CEUS) offers a clearer distinction between ablated and vital tissue [21]. Finally, our study's long‐term analyses are constrained by reduced statistical power resulting from patient attrition over the 5‐year follow‐up. To strengthen long‐term data specific to the Taiwanese population, future prospective, multicenter studies are warranted.

## 5 Conclusions

RFA demonstrates significant long‐term efficacy in reducing BTNs of various sizes, achieving high VRR with minimal complications. For larger nodules, long‐term monitoring using new growth as a detection parameter may be more effective than regrowth in determining recurrence and the need for retreatment.

## Conflicts of Interest

The authors declare no conflicts of interest.

## Data Availability

The data that support the findings of this study are available on request from the corresponding author. The data are not publicly available due to privacy or ethical restrictions.
